# 通过式固相萃取净化-超高效液相色谱-串联质谱法测定液体乳中10种氨基甲酸酯类农药残留

**DOI:** 10.3724/SP.J.1123.2023.03017

**Published:** 2023-09-08

**Authors:** Chao YUE, Chaoqun ZHAO, Sihao MAO, Zhanhua WANG, Bei SHI, Xinfeng XU, Jingjing LIANG

**Affiliations:** 1.浙江省食品药品检验研究院, 国家市场监管重点实验室(功能食品质量与安全领域), 浙江省市场局重点实验室(保健食品质量安全重点实验室), 浙江 杭州 310052; 1. Zhejiang Institute of Food and Drug Control, Key Laboratory of Functional Food Nutrition and Quality Safety for State Market Regulation, Key Laboratory of Health Food Quality Safety of Provincial Market Regulation, Hangzhou 310052, China; 2.浙江中医药大学, 浙江 杭州 310056; 2. Zhejiang Chinese Medical University, Hangzhou 310056, China

**Keywords:** 增强型基质脂质去除, 通过式固相萃取净化, 超高效液相色谱-串联质谱, 氨基甲酸酯类农药, 液体乳, enhanced matrix removal-lipid cleaning, pass-through solid-phase extraction purification, ultra performance liquid chromatography-tandem mass spectrometry (UPLC-MS/MS), carbamate pesticide, liquid milk

## Abstract

建立了应用直接通过式的增强型基质脂质去除柱联合超高效液相色谱-串联质谱法快速测定乳品中10种氨基甲酸酯类农药残留的分析方法。样品采用乙腈为提取液,沉淀除去蛋白质后,上清液通过Captiva EMR-Lipid柱净化,使用ACQUITY UPLC BEH C_18_柱(100 mm×2.1 mm, 1.7 μm)进行分离,以甲醇-0.1%甲酸水溶液为流动相进行梯度洗脱,流速0.3 mL/min,柱温35 ℃,采用电喷雾正离子模式(ESI^+^)和多反应监测(MRM)扫描方式检测,基质标准曲线外标法定量分析。结果显示,10种氨基甲酸酯类农药在2~200 μg/L范围内线性关系良好,相关系数(*r*)>0.999,方法的检出限为0.045~0.23 μg/kg,定量限为0.15~0.77 μg/kg;在空白基质中添加3个水平(15、50、100 μg/kg)的10种氨基甲酸酯类农药,进行加标回收率和重复性试验,回收率为68.7%~93.3%,相对标准偏差(RSD)为1.8%~8.0%。由结果可知,本研究方法高效、便捷、准确,适用于乳品中10种氨基甲酸酯类农药的批量检测。将本研究结果与现行国标测定方法中的氨基柱和ENVI^TM^-18 SPE柱净化效果进行比较,结果表明,经过Captiva EMR-Lipid直接通过式柱净化后,涕灭威及其代谢产物和甲萘威的回收率提升均在20%以上,本研究方法更适用于相对极性较强的氨基甲酸酯类农药,测定结果的重复性好,准确率高。

## Abstract (en)

Carbamates are used in broad-spectrum insecticides and herbicides, and have highly efficient, low-residue, and long-lasting characteristics. However, this type of pesticide exerts mutagenic, teratogenic, carcinogenic, and other adverse effects, and its frequent use can exceed the recommended scope and limits. Research on the determination of carbamate pesticides mainly focuses on foods of plant origin and pays less attention to foods of animal origin. The methods for carbamate determination described in the current national standards have complicated operating procedures and low efficiency. Therefore, highly efficient and accurate methods for carbamate detection in milk must be established. In this work, a rapid method based on pass-through solid-phase extraction (SPE) purification coupled with ultra performance liquid chromatography-tandem mass spectrometry (UPLC-MS/MS) was developed for the simultaneous determination of 10 carbamate pesticides in liquid milk. The pretreatment and instrument methods were systematically optimized. The milk sample was extracted with acetonitrile, and then purified using a Captiva EMR-Lipid filtration kit. The purified extract was separated on an ACQUITY UPLC BEH C_18_ column with mobile phase of methanol and 0.1% formic acid aqueous solution in gradient elution. The flow rate was 0.3 mL/min. Column temperature was 35 ℃. Quantitative analysis was performed using the external standard method with matrix matching curves. The 10 carbamate pesticides showed good linear relationships in the mass concentration range of 2-200 μg/L, with correlation coefficients greater than 0.999. The limits of detection (LODs) and quantification (LOQs) for the 10 carbamate pesticides were 0.045-0.23 and 0.15-0.77 μg/kg, respectively. Recovery tests were conducted using the blank-matrix method at three spiked levels of 15, 50, and 100 μg/kg, and good recoveries for the 10 carbamate pesticides were obtained. In particular, the recoveries for the three spiked levels of 15, 50, and 100 μg/kg were 68.7%-93.3% with relative standard deviations (RSDs) of 1.8%-8.0%. The proposed method is efficient, convenient, accurate, and suitable for the rapid detection of the 10 carbamate pesticides in liquid milk. Compared with the conventional NH_2_ and ENVI^TM^-18 SPE columns used in the national standard determination method, the proposed method demonstrated better purification effects. The recoveries for aldicarb sulfoxide, aldicarb sulfone, methomyl, and carbaryl after purification using the Captiva EMR-Lipid kit increased from 60% to 80%. Thus, the proposed method is suitable for targets with strong polarity and gives measurement results with good repeatability and accuracy.

氨基甲酸酯类农药是一种新型的广谱杀虫剂和除草剂,有高效、低残留、持续期长等特点,在农、林、牧业广泛应用^[1,2]^,但此类农药具有致突变、致畸、致癌等作用,且在使用过程中存在超范围、超限量使用的可能^[3,4]^,该类农药残留存在的食品安全风险值得注意。氨基甲酸酯类农药的测定研究多集中在植物源食品中^[5⇓⇓⇓⇓-10]^,动物源性食品涉及较少。涉及动物源性食品的国标^[11⇓⇓-14]^中,氨基甲酸酯类农药的测定方法多为液相色谱-质谱法,净化方法为固相萃取柱净化,净化柱有氨基柱、C_18_柱、中性氧化铝柱、凝胶柱等,净化步骤中包括固相萃取柱活化、洗脱、浓缩、复溶等步骤,操作复杂,耗时长。且此类农药具有种类多、极性强、热稳定性差等特点,操作过程中人为因素引起的误差大,增加了其准确定量的难度。QuEChERs和固相萃取法(SPE)近年在农残检测中应用广泛^[6,7,9,10]^,其操作灵活、简便、快速,但去除脂肪等物质能力较弱,净化效果不理想。

乳制品是居民膳食中的重要部分,近年来乳制品的质量安全引起广泛关注。乳品的污染来源较多,奶牛的饲料、饮用水、生长环境,生乳的存储、加工等环节均会存在农药污染风险。依据目前的研究报道可知,饲料^[15,16]^、土壤^[16⇓-18]^、地表水^[19,20]^中均有氨基甲酸酯类农药检出,其中克百威、甲萘威等检出率较高。在食物链与生物富集作用下,氨基甲酸酯类农药暴露对人体健康存在风险,尤其对乳品摄入量需求较高的婴幼儿群体,影响更加深远。液体乳类中的巴氏杀菌乳、酸乳等,由于加热温度低、时间短,农药受破坏程度低,其农药残留风险更高。目前乳品的质量安全研究多集中在兽药残留^[21⇓⇓⇓-25]^,农药残留研究相对较少,且现有农药残留分析的报道多集中在有机氯、有机磷、菊酯等^[26⇓-28]^,对于氨基甲酸酯这类新型农药的研究较少,且现有研究主要集中于克百威、灭多威等少量化合物。乳及乳制品中化学成分复杂,特别是蛋白质、脂肪、磷脂、糖类等大分子化合物,在实际的检测过程中影响此类农药残留物质检测结果的准确性。

增强型基质脂质去除净化材料(EMR-Lipid)是一种新型的高聚物吸附剂,此产品通过空间位阻和亲水性的协同作用,对C5及以上碳链的化合物具有极强的选择性,去除脂质的同时不吸附目标物^[29,30]^,该技术在动物源性食品的兽药残留、环境污染物等方面的检测上已得到广泛应用^[31⇓⇓⇓-35]^。Captiva EMR-Lipid通过型净化柱是在EMR-Lipid基础上升级成的固相萃取柱,可实现一步式通过净化。与常规的SPE相比,此净化柱可实现提取液直接上柱净化,操作步骤简便、快捷,批量检验的应用前景大。因大部分农药为极性物质,去除基质干扰物质时易造成目标物损失,依据EMR-Lipid材料的净化特点和氨基甲酸酯类农药的结构特点,本研究通过优化供试品提取净化方式和液相色谱体系,建立了Captiva EMR-Lipid通过型净化柱联合超高效液相色谱-串联质谱法测定液体乳中10种氨基甲酸酯类农药残留量的方法,以期为液体乳及乳制品的安全监管提供技术支撑。

## 1 实验部分

### 1.1 仪器与试剂

Shimadzu LC-30AD超高效液相色谱仪(日本岛津公司)串联AB-5500 QTRAP质谱仪,配有电喷雾离子(ESI)源(美国AB SCIEX公司); Milli-Q Gradient超纯水仪(法国Millipore公司);高速冷冻离心机(美国Thermo Fisher公司), XPE-205电子天平(瑞士Mettler公司), 0.22 μm有机滤膜(上海安谱实验科技有限公司)。

涕灭威亚砜(aldicarb sulfoxide)、涕灭威砜(aldicarb sulfone)、灭多威(methomyl)、3-羟基克百威(3-hydroxy carbofuran)、涕灭威(aldicarb)、速灭威(metolcarb)、克百威(carbofuran)、甲萘威(carbaryl)、异丙威(isoprocarb)、仲丁威(fenobucarb)标准品均来自上海安谱实验科技有限公司,质量浓度为100 mg/L。实验用水为超纯水,甲醇(MS级,德国默克公司),甲酸铵、甲酸(MS级,Sigma公司),乙腈、丙酮、正己烷(AR级,国药集团)。Captiva EMR-Lipid柱(200 mg/6 mL, Agilent公司)。

17批试验样品为市售预包装液体乳。

### 1.2 标准溶液的制备

分别精密吸取上述10种标准溶液0.50 mL于10 mL容量瓶中,加甲醇并稀释至刻度线,摇匀,取此稀释液2.0 mL于10 mL容量瓶中,加甲醇并稀释至刻度线,摇匀,得到质量浓度为1000 μg/L的混合标准工作溶液。以空白基质溶液为溶剂,配制得到2~200 μg/L的系列基质混合标准工作溶液。

### 1.3 供试品溶液的制备

称取液体乳样品5 g于25 mL容量瓶中,加乙腈稀释至刻度线并混合均匀,置于-18 ℃环境放置15 min,取出后将溶液以10000 r/min离心5 min,取上清液恢复至室温后直接加到Captiva EMR-Lipid柱上,收集后半段流出液适量,过0.22 μm有机滤膜,待测。

### 1.4 色谱条件

Waters ACQUITY UPLC BEH C_18_色谱柱(100 mm×2.1 mm, 1.7 μm)色谱柱;柱温35 ℃,流速0.3 mL/min,进样体积2 μL。流动相A为0.1%甲酸水溶液,流动相B为甲醇;梯度洗脱程序:0.01~1.5 min, 5%B~10%B; 1.5~5 min, 10%B~50%B; 5~8 min, 50%B~60%B; 8~10 min, 60%B~5%B; 10~12 min, 5%B。

### 1.5 质谱条件

ESI源,离子源温度500 ℃,离子化方式为正离子模式,多反应监测(MRM)模式,电喷雾电压5500 V,雾化气压力1.72×10^5^ Pa,气帘气压力2.04×10^5^ Pa,辅助器压力1.72×10^5^ Pa,其余质谱参数见表1。

**表 1:** 10种氨基甲酸酯类农药的保留时间及质谱参数

Compound	t_R_/ min	Precursor ion (m/z)	Daughter ion (m/z)	DP/ V	CE/ V
Aldicarb sulfoxide	4.03	207.1	132.0^*^	55	9
			89.2		20
Aldicarb sulfone	4.47	210.1	118.0^*^	30	17
			166.1		16
Methomyl	4.64	163.1	88.0^*^	38	12
			106.3		14
3-Hydroxy carbofuran	5.41	238.1	181.1^*^	70	16
			163.1		18
Aldicarb	6.15	208.1	116.1^*^	20	11
			89.3		25
Metolcarb	6.41	166.1	109.3^*^	80	16
			20.4		23
Carbofuran	6.74	222.1	165.1^*^	70	16
			123.3		29
Carbaryl	6.91	202.1	145.3^*^	100	17
			127.1		42
Isoprocarb	7.27	194.1	137.5^*^	110	12
			94.5		21
Fenobucarb	7.86	207.9	94.7^*^	90	23
			151.5		43

* Quantitative ion. DP: declustering potential; CE: collision energy.

## 2 结果和讨论

### 2.1 实验条件的优化

#### 2.1.1 质谱条件的优化

采用直接进样方式分别取0.1 mg/L的10种氨基甲酸酯类农药单一标准溶液注入离子源中,在正、负离子模式下进行母离子全扫描,分别获得目标化合物的分子离子峰。以每种目标化合物的准分子离子峰为母离子,进行二级质谱扫描,得到碎片离子信息,然后再对得到的每种目标化合物的二级质谱参数(如锥孔电压、碰撞能量等)进行优化。最后在MRM模式下优化离子源温度、去溶剂气温度、流速等质谱参数,最终优化参数见表1。

#### 2.1.2 液相色谱条件的优化

实验考察了甲醇、乙腈、甲酸铵水溶液、甲酸水溶液分别组合为流动相时目标物色谱峰的分离度和响应。结果显示,有机相为甲醇和乙腈均可以实现10种目标物的分离,但是乙腈为有机相时,涕灭威亚砜色谱峰出现裂缝且拖尾严重。水相中加入甲酸铵和甲酸时,10种目标物色谱峰的峰形、响应有明显改善和提高,两种流动相均呈弱酸性,更有利于其得到H变成[M+H]^+^在正离子模式下检测,且对目标物的离子化效率无差别。但甲酸水溶液配制更简单,且色谱柱平衡时间短,实验最终选择甲醇-0.1%甲酸水溶液为流动相。

实验分别考察了采用Waters Atlantis T3色谱柱(100 mm×2.1 mm, 1.7 μm)、Waters ACQUITY UPLC BEH C_18_色谱柱(100 mm×2.1 mm, 1.7 μm)、Agilent Zorbax C_18_色谱柱(100 mm×2.1 mm, 1.8 μm)对10种氨基甲酸酯类农药的分离效果。结果显示,Waters ACQUITY UPLC BEH C_18_色谱柱分离效果好,出峰时间适宜,因此选择该柱,相应的MRM色谱图见图1。

**图 1:**
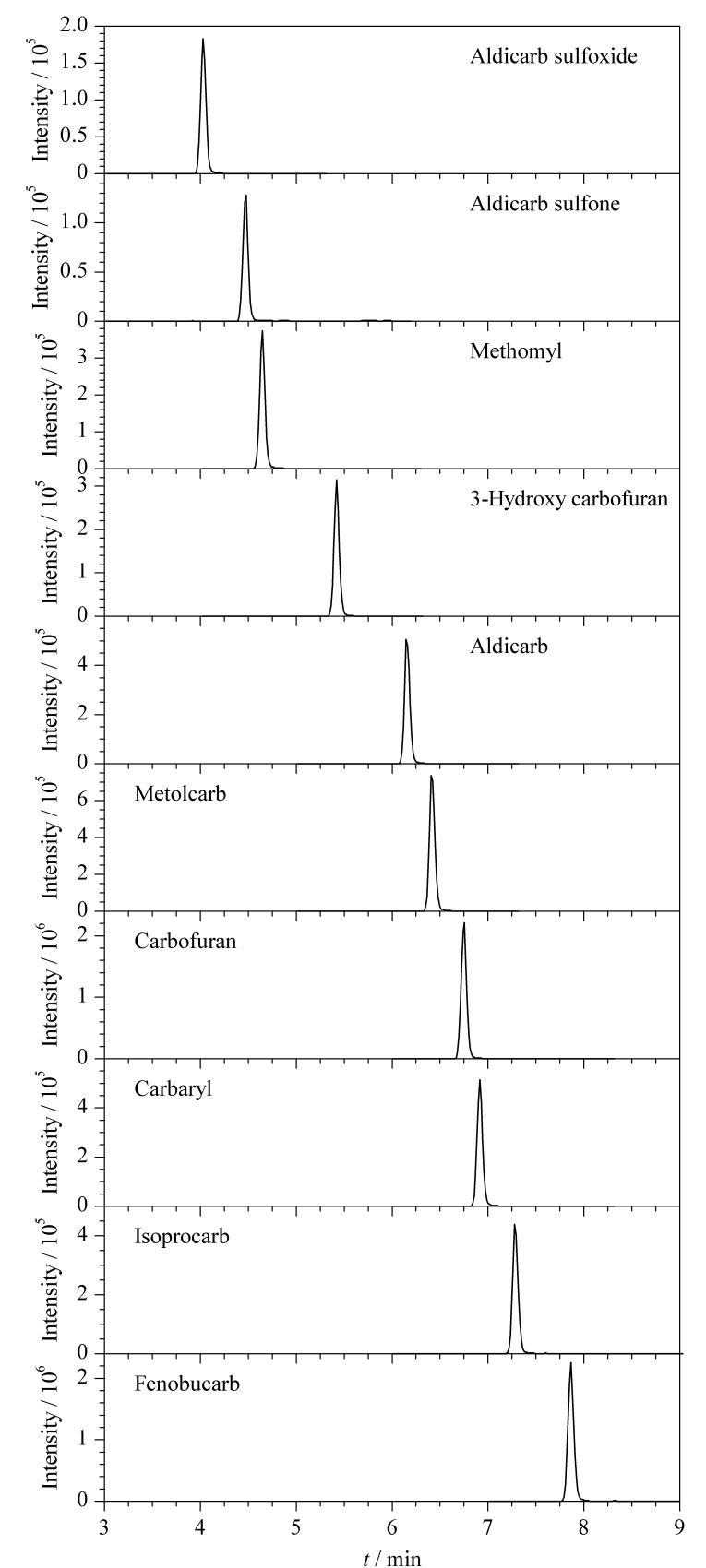
10种氨基甲酸酯类农药(50 μg/L)的MRM色谱图

#### 2.1.3 提取溶剂的选择

氨基甲酸酯类农药为中等极性农药,依据溶剂的相似相溶原理选择极性溶剂提取。样品中脂肪和蛋白质的去除是净化的关键步骤。实验考察了乙腈、丙酮、正己烷、甲醇为提取溶剂时沉淀蛋白质的效果,观察相同条件下静置后上清液的浑浊程度和通过微孔滤膜的难易程度,评价沉淀蛋白质的效果。结果显示,沉淀效果乙腈>丙酮>甲醇>正己烷,考虑提取液后续进入液相色谱-质谱联用仪测定,乙腈和甲醇溶剂更适用于检测系统。相较于甲醇,乙腈沉降蛋白质的效果更好,对不同极性目标物的提取效果更好,且对有机酸、糖、维生素等物质的溶解性低,萃取出的脂类杂质少^[35,36]^。实验同时比较了室温和-20 ℃条件下放置15 min时沉淀蛋白质的效果,可知低温更有利于蛋白质的沉淀。实验最终选择乙腈为提取溶剂,低温条件下沉淀蛋白质。

移取待测样品5 mL(为方便,条件考察时量取体积),分别添加5、10、15、20、25、30 mL乙腈,考察不同体积乙腈提取液的提取和净化效果,以10种目标物的加标回收率结果为评价指标。结果显示乙腈添加量为5 mL和10 mL时,蛋白质沉淀效果差,影响净化液流出速度和加标回收率结果;加入15 mL及以上时,提取净化效果最优。综合净化柱填料的适用性、方法检出限要求、待测样品的基质效应等因素考虑,实验选择加入约20 mL乙腈(乙腈体积占比约80%),即5 g样品用乙腈定容至25 mL。

#### 2.1.4 净化柱的选择

实验比较了Captiva EMR-Lipid柱和氨基(NH_2_)柱(200 mg/6 mL, Waters公司)、ENVI^TM^-18 SPE柱(200 mg/6 mL, Supelclean公司)的净化效果,以10种目标化合物的加标回收率结果为考察指标,结果见图2。

**图 2:**
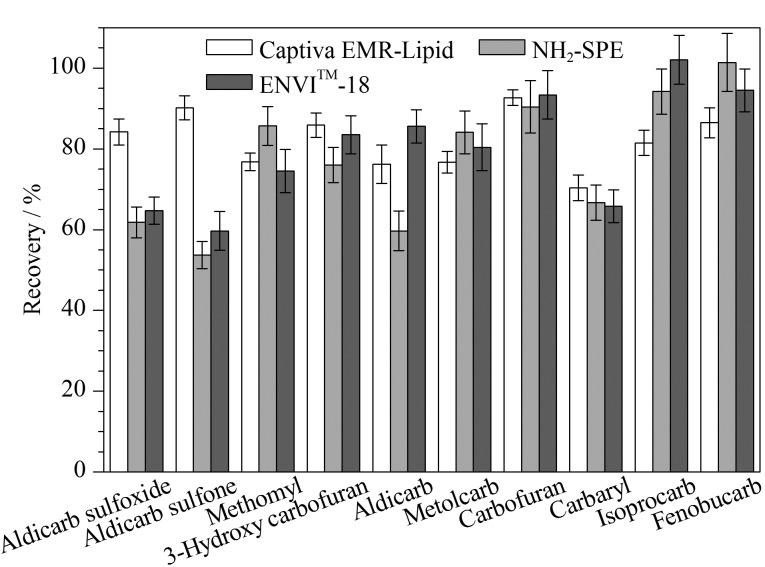
不同净化柱对10种氨基甲酸酯类农药回收率的影响(*n*=6)

氨基柱:吸取上清液5 mL, 40 ℃下氮气吹干,加入5 mL二氯甲烷-甲醇(99∶1, v/v)溶解。溶解液上柱,上样液流出近干后,使用20 mL二氯甲烷-甲醇(99∶1, v/v)洗脱,全部洗脱液于40 ℃下氮气吹干,加1 mL乙腈溶解,过0.22 μm有机滤膜,待测。

ENVI^TM^-18 SPE柱:吸取上清液5 mL, 40 ℃下氮气吹干,加入5 mL甲醇溶解。溶解液加在事先活化的ENVITM^TM^-18 SPE柱上,样液流出近干后,加20 mL甲醇溶液洗脱,全部洗脱液于40 ℃下氮气吹干,加1 mL乙腈溶解,过0.22 μm有机滤膜,待测。

Captiva EMR-Lipid柱:上清液直接上柱,收集后半段流出液适量,过0.22 μm有机滤膜,待测。

结果显示:经过Captiva EMR-Lipid柱净化后,目标物的回收率结果均>70%;氨基柱净化后涕灭威及其代谢物的回收率低,考虑可能原因是氨基柱对极性较强的氨基甲酸酯类物质的吸附力强,无法完全洗脱,导致目标化合物在净化过程中流失;对于极性较弱的异丙威、仲丁威物质,氨基柱(101%)和ENVI^TM^-18 SPE柱(94.5%)的加标回收率结果均优于Captiva EMR-Lipid柱(86.5%),但Captiva EMR-Lipid柱操作简便,其回收率结果的SD范围小。综上,Captiva EMR-Lipid柱净化效果可以达到国标方法的净化要求,且更适用于极性较强的涕灭威类物质的检测,操作过程简便,结果重复性好。

### 2.2 基质效应

用初始比例流动相溶液配制20 μg/L的10种氨基甲酸酯类农药标准溶液,用空白基质溶液配制20 μg/L的基质混合标准溶液。以两者对应氨基甲酸酯类农药的峰面积比值乘以100%作为基质效应的评估值^[37⇓-39]^。结果显示,不同氨基甲酸酯类农药的基质效应为35.7%~67.5%,存在中等和强基质效应。因此,实验采用基质匹配标准曲线,以降低基质效应对目标化合物定量的影响。

### 2.3 方法学考察

#### 2.3.1 线性范围、检出限和定量限

将1.2节配制的系列基质混合标准工作溶液进样测定,以10种目标化合物的质量浓度为横坐标,以对应的峰面积为纵坐标绘制标准曲线并计算回归方程及相关系数。采用空白基质加标并逐级稀释的方式进行检出限(LOD)和定量限(LOQ)的测定。以信噪比(*S/N*)≥3确定10种农药的检出限,以*S/N*≥10确定10种农药的定量限。10种农药的线性范围、校准曲线方程、相关系数、LOD和LOQ结果见表2。相关系数均在0.999以上。

**表 2:** 10种氨基甲酸酯类农药的线性关系、检出限及定量限

Compound	Linear range/(μg/L)	Linear equation	r	LOD/(μg/kg)	LOQ/(μg/kg)
Aldicarb sulfoxide	2-200	y=6.92×10x+2.35×10^4^	1.0000	0.045	0.15
Aldicarb sulfone	2-200	y=1.08×10^4^x-1.40×10^4^	0.9996	0.046	0.15
Methomyl	2-200	y=3.50×10^4^x-5.99×10^4^	0.9996	0.23	0.76
3-Hydroxy carbofuran	2-200	y=7.20×10^4^x+9.10×10^4^	0.9997	0.23	0.76
Aldicarb	2-200	y=1.48×10^5^x-4.40×10^5^	0.9993	0.045	0.15
Metolcarb	2-200	y=9.98×10^4^x+6.70×10^4^	1.0000	0.22	0.76
Carbofuran	2-200	y=7.33×10^4^x+3.96×10^5^	0.9999	0.23	0.76
Carbaryl	2-200	y=4.34×10^4^x+1.90×10^4^	0.9999	0.21	0.76
Isoprocarb	2-200	y=2.07×10^5^x-3.39×10^5^	1.0000	0.22	0.77
Fenobucarb	2-200	y=4.15×10^5^x+1.09×10^5^	0.9999	0.23	0.76

y: peak area; x: mass concentration, μg/L; r: correlation coefficient.

与使用质谱方法检测的国家标准相比较,本研究建立方法的检出限和定量限结果均满足现有国标要求。GB 23200.90-2016中10种目标物的定量限均为0.01 mg/kg, GB 31658.10-2021中10种目标物的检出限为0.5 μg/kg,定量限为1.0 μg/kg。

#### 2.3.2 加标回收率和精密度

采用不含待测物的空白样品进行加标回收率试验,加标水平设置为3个,每个添加水平做6次平行,考察该方法的准确度和精密度,结果见表3。结果表明,本方法中10种氨基甲酸酯类农药的回收率为68.7%~93.3%,相对标准偏差(RSD)为1.8%~8.0%。表明该方法满足分析要求,可用于10种氨基甲酸类农药残留量的准确定性和定量。

**表 3:** 10种氨基甲酸酯类农药的加标回收率和RSD(n=6)

Compound	Added/ (μg/kg)	Found/ (μg/kg)	Recovery/ %	RSD/ %	Compound	Added/ (μg/kg)	Found/ (μg/kg)	Recovery/ %	RSD/ %
Aldicarb sulfoxide	15	13.3	88.7	6.5	Metolcarb	15	13.5	90.0	4.5
	50	42.1	84.2	5.9		50	38.4	76.7	5.1
	100	79.3	79.3	6.7		100	77.6	77.6	4.9
Aldicarb sulfone	15	13.0	86.7	3.9	Carbofuran	15	12.7	84.7	6.0
	50	45.1	90.2	4.8		50	46.4	92.7	5.1
	100	92.7	92.7	6.8		100	89.9	89.9	2.5
Methomyl	15	11.5	76.7	5.3	Carbaryl	15	10.9	72.7	4.8
	50	38.4	76.8	2.7		50	35.3	70.5	4.1
	100	74.2	74.2	4.6		100	68.7	68.7	8.0
3-Hydroxy carbofuran	15	12.1	80.7	4.0	Isoprocarb	15	13.0	86.7	3.9
	50	43.0	85.9	1.8		50	40.8	81.5	5.8
	100	83.1	83.1	2.6		100	84.6	84.6	4.8
Aldicarb	15	11.6	77.3	4.6	Fenobucarb	15	14.0	93.3	4.8
	50	38.1	76.2	4.6		50	43.3	86.5	3.5
	100	70.8	70.8	5.7		100	82.6	82.6	5.5

### 2.4 样品的测定

采用本研究建立的方法,对17批市售液体乳进行测定,结果显示均未检出10种氨基甲酸酯类农药。

## 3 结论

本研究通过优化提取方法和流动相体系,建立了应用Captiva EMR-Lipid直接通过式净化方法联合UPLC-MS/MS同时测定液体乳中10种氨基甲酸酯类农药的定性定量方法。该法快速高效、准确度好,灵敏度高,重复性好,可满足日常大批量检测的需求,适用于液体乳中10种氨基甲酸酯类农药的快速测定。同时为动物源食品中氨基甲酸酯类农药的测定提供新思路。
